# Microstructure and Microorganisms Alternation of Paddy Soil: Interplay of Biochar and Water-Saving Irrigation

**DOI:** 10.3390/plants14101498

**Published:** 2025-05-16

**Authors:** Jiazhen Hu, Shihong Yang, Wim M. Cornelis, Mairan Zhang, Qian Huang, Haonan Qiu, Suting Qi, Zewei Jiang, Yi Xu, Lili Zhu

**Affiliations:** 1 College of Agricultural Science and Engineering, Hohai University, Nanjing 211100, China; 2 Jiangsu Province Engineering Research Center for Agricultural Soil-Water Efficient Utilization, Carbon Sequestration and Emission Reduction, Nanjing 210098, China; 3 Department of Faculty of Bioscience Engineering, Ghent University, 9000 Ghent, Belgium; 4 Urban Water Scheduling and Information Management Department of Kunshan City, Kunshan 215300, China

**Keywords:** soil throat structure, microbial community composition, soil aggregates, soil physicochemical characteristics, irrigation, biochar

## Abstract

Biochar application and controlled irrigation (CI) enhance water conservation, lower emissions, and increase crop yields. However, the synergistic effects on the relationship between paddy soil microstructure and microbiome remain poorly understood. This study investigates the impact of different irrigation regimes and biochar applications on soil physicochemical properties, soil microstructure, and the composition and functions of soil microorganisms in paddy soil. The CA treatment (CI with 60 t/hm^2^ biochar) showed higher abundances of *Mycobacteriaceae*, *Streptomycetaceae*, *Comamonadaceae*, and *Nocardioidaceae* than the CK treatment (CI without biochar), which was attributed to two main factors. First, CA increased the pore throat equivalent radius (EqR), throat surface area (SAR), total throat number (TTN), volume fraction (VF), and connected porosity (CP) by 1.47–9.61%, 7.50–25.21%, 41.55–45.99%, 61.12–73.04%, and 46.36–93.75%, respectively, thereby expanding microbial habitats and providing refuges for microorganisms. Second, CA increased the cation exchange capacity (CEC), mean weight diameter (MWD), soil organic carbon (SOC), and total nitrogen (TN) by 22.14–25.06%, 42.24–56.61%, 22.98–56.5%, and 9.41–87.83%, respectively, reinforcing soil structural stability and carbon storage, which promoted microbial community diversity. FK (flood irrigation without biochar) showed no significant correlations with these environmental factors. Compared to CK soil metabolites at Level 2 and Level 3, FK exhibited higher levels of the citrate cycle, indicating that changes in water and oxygen environments due to CI reduced soil organic matter decomposition and carbon cycle. CA and CK strongly correlated with the soil microstructure (VF, CP, TTN, SAR, EqR), and CA notably enhanced soil metabolites related to the synthesis and degradation of ketone bodies, suggesting that biochar can mitigate the adverse metabolomic effects of CI. These results indicate that biochar application in CI paddy fields highlights the critical role of soil microstructure in microbial composition and function and better supports soil sustainability.

## 1. Introduction

Soil microbiomes drive key functions in agricultural ecosystems, determining soil fertility, crop productivity, and resilience [1]. The microbiome is closely linked to soil structure, including aggregates and pore connectivity, which regulate the flow of water, oxygen, and nutrients [2]. Rice is a staple food for more than half of the global population and accounts for approximately 20% of the world’s dietary energy supply [3]. Conventional rice irrigation practices (Flooding Irrigation, FI), which involve prolonged soil submergence, result in significant alterations to the soil’s physical, chemical, and biological properties compared to dryland soils [4]. In contrast, controlled irrigation (CI), a water-saving irrigation method in paddy fields, utilizes frequent wetting and drying cycles, which can induce soil mineralization, modify soil structure, and influence the composition and activity of soil microbial communities [5,6]. Consequently, the investigation of the correlation between soil structure and microbial function under varying paddy field irrigation regimes has emerged as a pivotal area of research.

Soil aggregates are essential units of soil structure, comprising minerals, organic matter, and microorganisms, and are particularly sensitive to tillage practices [2,7]. These aggregates are broadly classified into two functional categories based on their size and composition. Microaggregates (particles with a diameter of less than 250 µm) form through the binding of small particles by organic and inorganic materials, such as clay and carbonate crystals, and are stabilized by physical and chemical interactions, including flocculation and organic bonds [8]. In contrast, macroaggregates (>250 µm) are larger, more complex structures that encompass smaller aggregates and rely on biological processes—such as fungal hyphae and root networks—for stability [9]. Aggregate stability is a critical determinant of soil health and is heavily influenced by soil organic carbon (SOC) and total nitrogen (TN) levels, along with the carbon-to-nitrogen (C/N) ratio. SOC functions as a binding agent, thereby enhancing aggregate stability by supporting microbial growth and activity [10]. While nitrogen’s effects on aggregate stability can vary, combining N with organic inputs typically strengthens aggregate stability [11,12]. The C/N ratio plays a similarly pivotal role, as higher ratios often lead to more persistent improvements in aggregate stability [13]. Water-saving irrigation practices show mixed effects on SOC, N, and the C/N ratio, depending on factors such as soil type, irrigation method, and management [14]. The existing literature reports a range of outcomes, including positive correlations [15,16], negative correlations [17,18,19], and no significant correlation [20,21]. There is still a limited understanding of how the chemical distribution and pore structure of aggregates affect soil stability under different irrigation practices. Advancing sustainable soil management will require a deeper understanding of the interactions between aggregate stability, chemical composition, and microstructural pore characteristics across different irrigation conditions.

The structure and stability of soil aggregates are critical to the abundance, diversity, and activity of soil microbiota [22,23]. Aggregates of different sizes provide distinct habitats for microorganisms: around 90% of soil bacteria are associated with microaggregates [24], while fungi contribute to aggregate formation through hyphal networks [25]. Although separated by physical barriers and distance, microbial communities within and between aggregates can interact during soil wetting, facilitating nutrient, metabolite, and genetic exchanges [26]. Larger pores within and between macroaggregates favor the survival of aerobic bacteria, while smaller pores within microaggregates offer both aerobic and anaerobic niches through wet–dry cycles [27]. Different moisture levels, influenced by irrigation practices, affect crop physiology, soil nutrient availability, and microbial community composition [28,29]. Traditional tillage exposes deeper soil layers to surface wet–dry cycles, increasing macroaggregate turnover, disrupting existing pore networks, and accelerating SOC decomposition [30]. Thus, the functionality of soil microbial communities largely depends on the impact of environmental factors on soil aggregates and their micropore structure.

Biochar as a soil amendment is considered a promising strategy for enhancing soil fertility and carbon sequestration [22,31]. Biochar amendments affect soil structure and function, primarily by affecting nutrient cycling [32]. Biochar also affects soil microbial communities by altering various soil properties, including organic matter content, water-holding capacity, acidity, nutrient availability, or levels of potentially toxic substances [33]. Previous studies have combined biochar with CI to mitigate the adverse effects of CI-induced soil mineralization [5], enhance soil nutrient levels [34], increase soil organic matter stability [35], and improve soil microbial biomass [36]. However, the effects of biochar on soil microbial communities vary. For instance, biochar can increase bacterial abundance [37], reduce overall soil microbial diversity [38], or have minimal impact on soil microbial communities [39]. The combination of biochar and CI modifies soil structure, altering microbial habitats, but our understanding of these changes is still incomplete. X-ray computed tomography (CT) has become a critical tool for studying soil micropore structures, allowing non-destructive, detailed imaging of soil porosity, connectivity, and spatial network features [40,41]. These techniques aid in understanding the interactions between soil microstructure, microorganisms, and SOC components [42,43].

We hypothesize that a combination of biochar addition and irrigation mode will significantly impact the soil physicochemical properties and soil microstructure in paddy fields, thereby altering the composition and function of soil bacterial and fungi communities. This work aimed to (i) quantify the effects of biochar amendment and irrigation mode on soil microstructure, particularly the morphological characteristics of pore throats, using CT technology, (ii) investigate the relationship between soil microstructure changes and key metabolic functions using metabolomics, and (iii) assess how biochar application enhances soil microstructure stability, microbial community diversity, and functional metabolic capacities while mitigating the adverse impacts of CI on soil metabolism. In contrast to prior studies that primarily examined biochar’s effects on either microbiome or soil structure [32,33,34,35,36,37,38,39], this study pioneers an integrated assessment of how soil microstructure governs microbial composition and functionality across varying irrigation regimes and biochar applications, providing a scientific basis for future soil improvement and management strategies.

## 2. Results

### 2.1. Soil Physicochemical Characteristics

#### 2.1.1. Soil Basic Properties

**Figure 1:**
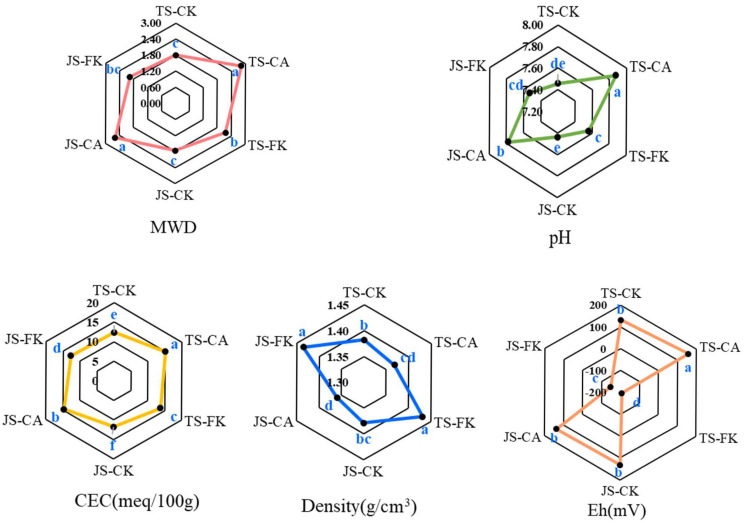
Basic properties of soil paddy under different irrigation and biochar management strategies. Mean weight diameter (MWD) of water-stable aggregates, pH, cation exchange capacity (CEC), density, and oxidation-reduction potential (Eh) content under different treatments. Different lowercase letters indicate a significant difference at *p* < 0.05 in comparisons of various treatments.

ANOVA results indicated that both biochar and irrigation significantly impacted soil MWD, pH, CEC, density, and Eh (*p* < 0.01), while the growth stages showed no significant effect (*p* > 0.05) (Table S1) (Figure 1). Compared to CK, the MWD of CA increased by 42.24–56.61% (*p* < 0.05), indicating that biochar amendment positively affects soil aggregation in paddy fields. In contrast, the MWD in FK was 11.80–20.29% higher than in CK, showing that biochar mitigates the negative impacts of CI on soil structure. Soil pH values for CK, FK, and CA were 7.44–7.46, 7.53–7.56, and 7.78–7.87, respectively. Compared to CK, CA significantly increased soil pH by 4.57–5.54%. CEC was significantly higher in CA compared to FK and CK across all stages, with FK showing an 8.17–11.72% increase over CK. Soil density was higher in FK than in CK, and CA significantly reduced soil density. The oxidation-reduction potential (Eh) values were 1.69–1.89 times lower in FK than in CK, indicating more anaerobic conditions under continuous irrigation.

#### 2.1.2. SOC, and TN in Bulk Soil and Aggregate Classes

**Figure 2:**
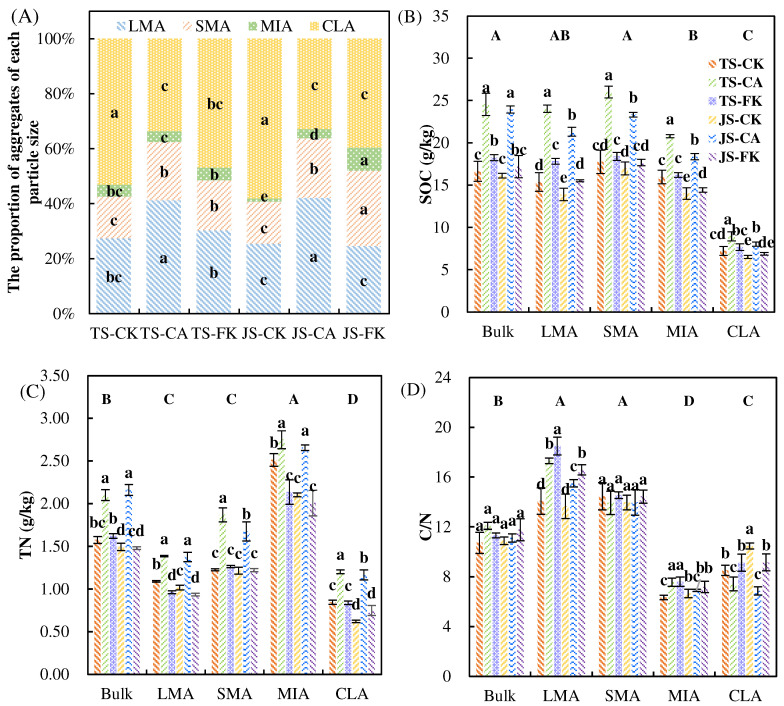
Soil aggregate classes and their characteristics in soil organic carbon (SOC), TN, and C/N under different water-carbon management strategies. Size distribution (**A**) of water-stable aggregate. LMA, large macroaggregates (>2000 μm); SMA, small macroaggregates (250–2000 μm); MIA, microaggregates (53–250 μm); CLA, silt and clay (<53 μm). Uppercase letters denote differences between the soil aggregate classes at *p* < 0.05 level. Lowercase letters indicate differences among the six isolated aggregate classes at *p* < 0.05 level. (**B**) SOC content across different soil aggregate sizes; (**C**) TN content across different soil aggregate sizes; (**D**) C/N content across different soil aggregate sizes.

The average values of soil aggregates followed the order CLA > LMA > SMA > MIA, among which LMA (31.86%) was 12.16% lower than that of CLA (44.02%) (Figure 2A). CLAs were the most common type of soil aggregate, accounting for 32.87–58.12% of all soils, while MIAs were the least common, accounting for 1.10–8.40%. At different growth periods, CA significantly boosted LMA and SMA (*p* < 0.05) by 50.63–65.67% and 39.24–41.28%, respectively, compared to CK, indicating that the biochar application can increase the proportion of >250 μm aggregates, leading to increased stability of soil aggregates. The MIA and SMA for FK are significantly higher than for CK, particularly during the JS, mainly due to the more frequent wet–dry cycles experienced under CI compared to FI. The ANOVA results indicate that both biochar and irrigation had significant effects on the concentrations of MWD, SOC, TN, C/N LMA, C/N MIA, and C/N CLA (Table S1). Compared to CK, CA significantly increased the SOC by 22.98–56.5% and TN by 9.41–87.83% across both bulk soil and various aggregate classes (*p* < 0.05), without significantly affecting the C/N ratio (Figure 2B–D). In LMA, the FK values for SOC and C/N were 11.64–15.94% and 21.44–31.46% higher than the CK values, respectively, while the FK values for TN were 8.20–11.78% lower than the CK values. Among the treatment groups, the SOC content was highest in SMA, whereas TN content was highest in MIA.

### 2.2. CT-Measured Microstructure Characteristics

#### 2.2.1. Pore Structure

Table 1 shows the volume fraction (VF), equivalent diameter (EqD), area, connected porosity (CP), and fractal dimension (FD) of soil samples extracted from the region of interest (ROI) using SR-μCT. Significant differences were observed in VF and CP, with the ANOVA results showing that both metrics were significantly influenced by biochar, irrigation, and their interactions (*p* < 0.05) (Table S1). Compared to CK, CA exhibited increases in VF and CP by 61.12–73.04% and 46.36–93.75%, respectively, which indicate that biochar enhances soil porosity and macroaggregate connectivity. Soils under FK exhibited reduced VF and CP compared to CK, likely due to prolonged submergence under FI, which saturates the soil pores and increases pressure. This compaction rearranges soil particles, leading to decreased porosity and connectivity. CA and CK showed significant differences across growth stages, with TS-CK < JS-CK and TS-CA < JS-CA indicating that as the growth period progresses, the frequency of wet–dry cycles in CI increases, leading to a gradual expansion in soil porosity.

**Figure 3:**
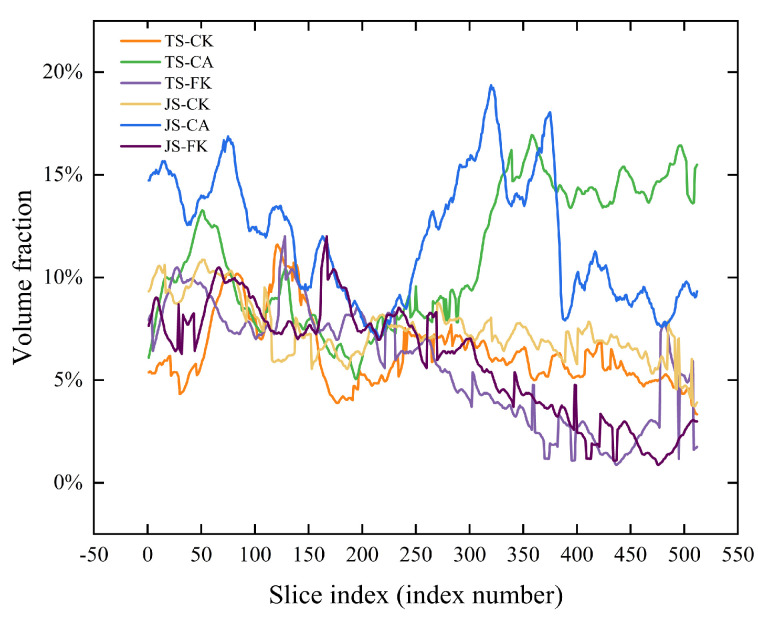
Volume fraction distribution in slices of soil sample. The volume fraction of the soil sample slices was based on the region of interest (ROI).

Analysis of the volume fraction distribution within the ROI slices shows that between slices 20–70 and 250–512, CA significantly increased pore volume fraction (Figure 3). In contrast, the CK and FK treatments displayed a decreasing trend in pore volume fraction as soil depth increased. Notably, between slices 300–470, the pore volume fraction was consistently higher in CK than in FK at different growth stages, with CK exhibiting a more complex internal structure and more uniform pore distribution.

#### 2.2.2. Pore Throat Structure

The total throat number (TTN), throat surface area (SAR), equivalent radius (EqR), and channel length (CL) were calculated using Avizo software (Table 2). Compared to the FK treatment, the CA and CK treatments significantly increased the TTN, SAR, and EqR. However, the average CL showed no significant variation across treatments. During different growth stages, the TTN of CA was 45.99% higher at the TS and 41.15% higher at the JS than under CK (*p* < 0.05). This increase can be attributed to biochar’s rich pore throat structure, which enhances soil particle aggregation, forming more stable soil aggregates and boosting both the number of throats. Conversely, the TTN in the FK treatment was lower by 71.72% at TS and 69.06% at JS, compared to the CK treatment. This reduction may result from the repeated wetting and drying cycles under CI, which cause soil expansion and contraction, creating tension between aggregates and reducing the total throat number. Furthermore, SAR and EqR were significantly higher in JS-CA than in JS-CK, with increases of 25.21% and 9.61%, respectively.

**Figure 4:**
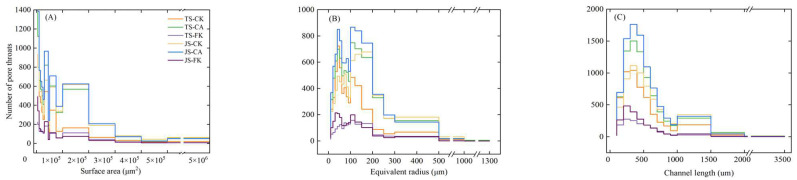
Distributions of surface area (**A**), equivalent radius (**B**), and channel length (**C**) of pore throats in the ROI of the sample under different water-carbon management strategies.

Figure 4 illustrates the relationship between pore throat number and SAR, EqR, and CL under various treatments. The pore throat number regions with SAR (<1 × 10^5^ μm^2^), EqR (<200 μm), and channel length (<700 μm) have the highest distribution, with significant differences observed among the treatments. Compared to FK, both CA and CK show a higher number of throats, with the difference being most prominent during the JS (Figure 4A). The EqR values in CA and CK are consistently higher in the 10–40 μm and 60–150 μm ranges compared to in FK (Figure 4B). Specifically, the EqR values for CA (6.65–1264.59 μm) are higher than those for CK (6.65–1183.95 μm) and FK (7.55–840.47 μm), indicating that CI and biochar application have a significant impact on soil throat structure. The CL ranges from 54.98–3200.15 μm in CK, 51.49–2941.24 μm in CA, and 66.20–2184.60 μm in FK (Figure 4C). Compared to CK, the number of throats with CL between 300–700 μm is higher under CA but lower under FK. A comparison between TS-FK and JS-FK reveals that the throat number within different CL ranges of variation is small, primarily due to the minimal changes in soil structure under prolonged FI conditions.

### 2.3. Composition and Functional Properties of Soil Bacterial and Fungi Communities

#### 2.3.1. Community Structure of Bacterial and Fungi Genes

Biochar application and irrigation patterns altered the microbial community structure in paddy soil. Microbial diversity was assessed using the Chao1, Simpson, and Shannon indices (Figure S1). The Chao1 and Shannon indices for bacteria exceeded those of fungi, indicating greater bacterial species richness and a more balanced distribution of species richness in paddy soil. The Simpson index showed no significant differences between treatments in bacterial and fungal communities. The Chao1 index for soil bacteria was significantly lower in CK compared to FK (*p* < 0.05), with no significant difference between CK and CA during the TS (Figure S1A). This may be attributed to the frequent fluctuations in moisture and oxygen conditions in CI paddy fields, which inhibit the physiological functions of certain bacterial communities, thereby reducing soil community diversity. In this study, the Chao1 index of fungal communities across treatments ranged from 61–99.6, and the Shannon index ranged from 2.10–2.86 (Figure S1B,F). Compared to FK, the Chao1 index of fungal communities in CK was 34.79% (TS) and 29.68% (JS) lower, while the Shannon index in CK was significantly reduced by 17.26% (TS).

**Figure 5:**
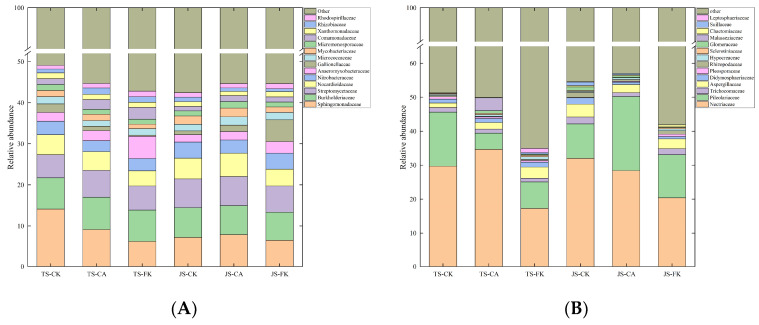
Relative abundance of the top 15 family level bacteria (**A**) and fungi (**B**) with the highest abundance.

The relative abundances of bacteria and fungi at the family (Figure 5) and phyla (Figure S2) levels were analyzed for various treatments and growth stages (top 15). The fungal community structure varied significantly with the rice growth stages, especially with *Pileolariaceae* being markedly lower in JS-CK compared to other treatments, while the bacterial community structure remained relatively stable. At the family level, the bacterial communities across different treatments were predominantly composed of *Sphingomonadaceae* (6.22–14.11%), *Burkholderiaceae* (6.82–7.87%), and *Streptomycetaceae* (5.64–7.11%), while the fungal communities were mainly composed of *Nectriaceae* (17.25–34.65%), *Pileolariaceae* (4.76–21.78%), and *Aspergillaceae* (1.30–3.79%). The CA abundance of *Sphingomonadaceae*, *Streptomycetaceae*, *Nocardioidaceae*, and *Mycobacteriaceae* was higher by 9.30%, 1.71%, 11.61%, and 5.10%, respectively, compared to CK, during the JS (Figure 5A). This increase is likely due to their preference for aerobic conditions and neutral to slightly alkaline soils, with biochar application increasing the soil pH. The abundance of *Sphingomonadaceae* in TS-CK and TS-CA is significantly lower than in JS-CK and JS-CA, decreasing by 6.90% and 1.24%, respectively. The high carbon content during the TS likely provided abundant carbon resources for the growth of *Sphingomonadaceae*, promoting their metabolic activities (Figure 2). In FK, *Anaeromyxobacteraceae* abundance was 1.05–3.24% higher than in CK, likely due to their preference for low-oxygen environments. *Nectriaceae* play a crucial role in the decomposition of organic matter and nutrient cycling but also threaten crop health and yield as plant pathogens. This study found that the abundance of *Nectriaceae* in CK was significantly higher than in FK, by 12.44% (TS) and 11.61% (JS), respectively (Figure 5B).

At the phyla level, *Proteobacteria* (32.27–40.69%), *Actinobacteria* (16.81–24.21%), and *Acidobacteria* (1.01–1.50%) were the dominant bacterial in all treatments (Figure S2A). In this study, *Actinobacteria* exhibited the CA > CK > FK under different periods, indicating that biochar increases the activity of microbes involved in the carbon cycle. Compared to CK, CA reduced the relative abundance of *Acidobacteria* by 0.14–0.39%. This reduction can be attributed to biochar-induced increases in soil pH (Figure 1), as *Acidobacteria* thrive in low pH environments and are sensitive to pH elevations. *Ascomycota* (28.49–44.90%) and Basidiomycota (6.69–25.43%) are the dominant fungi (Figure S2B). The abundance of *Ascomycota* in CK was significantly higher than in FK, increasing by 8.25–14.02%. This difference is likely attributable to the fact that FI significantly reduces soil oxygen content, creating an anaerobic environment that is unfavorable for the growth and metabolism of aerobic *Ascomycota*. *Ignavibacteriae* was exclusively present in TS-FK and JS-FK treatments, likely due to its preference for anaerobic or microaerophilic environments, which are more common under flooding conditions.

#### 2.3.2. Functional Categories of Microorganisms

KEGG pathway enrichment analysis was used to study the functional content of paddy soil bacterial communities under different irrigation and biochar management strategies and how changes in these microbial communities affect metabolic functional diversity. The Level 1 pathway abundance analysis identified six metabolic pathways, with metabolism being the most abundant (73.03–73.76%), followed by genetic information processing (11.11–11.45%), cellular processes (5.09–5.53%), human diseases (4.60–4.88%), organic systems (3.10–3.31%), and environmental information processing (1.97–2.07%) (Table S2). There were significant differences in Cellular Processes between CK and FK at different stages, with FK showing an increase of 4.80–7.80% compared to CK. The TS-FK and JS-FK samples exhibited significant clustering, indicating that the KEGG pathway expression profiles of these samples are similar and display high levels of expression (Figure S3). These included most metabolic pathways, such as glycan biosynthesis and metabolism, energy metabolism, amino acid metabolism, metabolism of terpenoids and polyketides, metabolism of other amino acids, global and overview maps, carbohydrate metabolism, biosynthesis of other secondary metabolites, metabolism of cofactors and vitamins, nucleotide metabolism, xenobiotics biodegradation and metabolism, and lipid metabolism. These metabolic pathways encompass the fundamental life activities of cells, including energy conversion, synthesis and decomposition of substances, and signal transduction.

**Figure 6:**
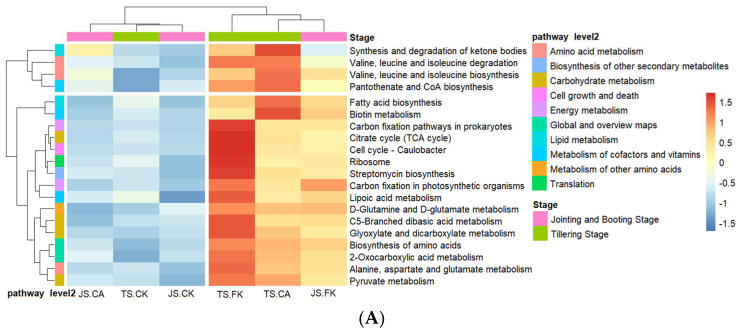

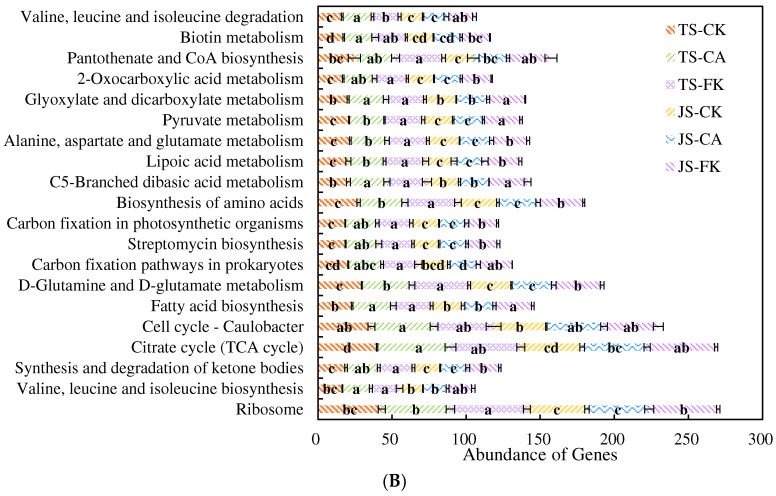
Gene abundance of KEGG metabolic pathways: cluster heatmap of Level 2 and Level 3 (**A**), metabolic pathways top 20 abundance of Level 3 enriched (**B**). Different lowercase letters indicate significant differences at the 0.05 level in the same genes.

Figure 6A shows that each sample of Level 3 was divided into four clusters: the first type was JS-CA; the second type was TS-CK, JS-CK; the third type was TS-FK; the fourth type was TS-CA and JS-FK. Among the top 20 KEGG Level 3 metabolites information, amino acid metabolism accounted for 3, carbohydrate metabolism for 4, and metabolism of cofactors and vitamins for 3 (Figure 6A). During different growth stages, the citrate cycle (TCA cycle) showed FK > CK and CA > CK, indicating that changes in water and oxygen environments caused by CI reduced soil organic matter decomposition and disrupted the carbon cycle, but the use of biochar could mitigate this effect (Figure 6B).

## 3. Discussion

### 3.1. Influence of Soil Physicochemical Properties on Soil Microstructure Under Biochar and Water-Saving Irrigation

**Figure 7:**
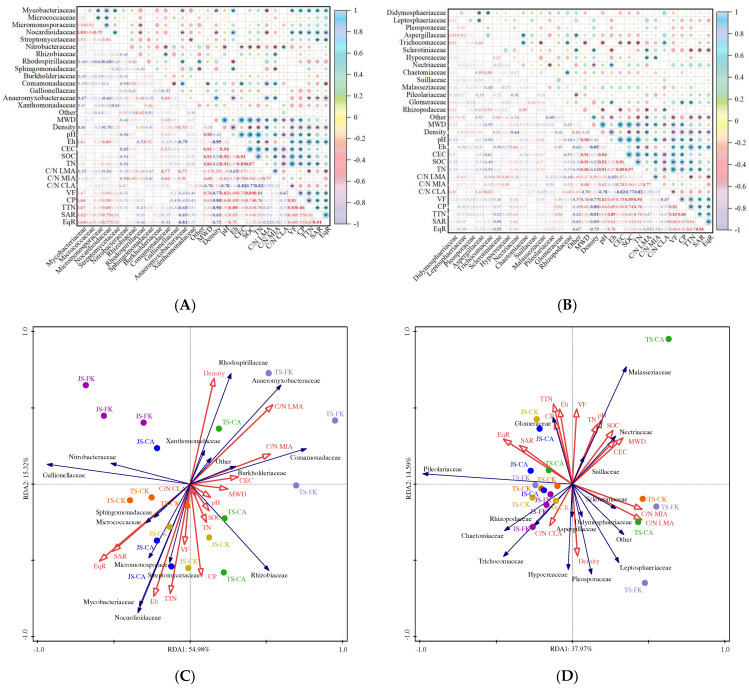
Pearson correlation coefficients of the relationships between dominant bacterial families (**A**) and fungal families (**B**), and soil physicochemical properties (MWD, density, pH, Eh, CEC, SOC, TN, C/N LMA, C/N MIA, C/N CLA) and soil microstructure (VF, CP, TTN, SAR, EqR), respectively (* *p* < 0.05). Redundancy analysis (RDA) curves of the dominant bacteria family (**C**), and fungi family (**D**). Red arrows indicate environmental variables; blue arrows indicate species; points represent the different treatment soil samples. * *p* < 0.05.

Soil aggregates are fundamental components of soil structure, formed by the aggregation of soil particles [2]. This study found that MWD was significantly positively correlated with pH (0.90), CEC (0.91), SOC (0.91), and TN (0.86) (Figure 7A). Several factors explain these relationships: Firstly, soil pH value can promote the decomposition of soil organic matter and the release of nutrients (with a correlation coefficient of 0.92 between pH and SOC), thereby enhancing soil structural stability [44]. Secondly, the increased CEC provides negatively charged adsorption sites, which facilitate the formation of stronger soil aggregates by improving their mechanical integrity [45]. Lastly, higher SOC and TN levels boost microbial biomass and activity, promoting the production of binding agents such as polysaccharides that stabilize aggregates and increase MWD [46,47]. At various growth stages, CA significantly raised the proportion of aggregates (>250 μm), leading to a 42.24–56.61% increase in MWD. This was due to significant increases in C/N LMA and C/N MIA under CA treatment, which increased by 13.54–23.04% and 3.72–19.22%, respectively, while the C/N CLA decreased by 12.77–34.51% (Figure 2). Furthermore, MWD showed a positive correlation with C/N LMA and C/N MIA but a negative correlation with C/N CLA (Figure 7), suggesting that biochar application enhances soil stability under CI conditions. Our findings align with Situ et al. [34], who reported that biochar additions across four consecutive rice seasons increased the proportion of water-stable aggregates (>250 µm) and progressively raised MWD. However, contrasting findings from Grunwald et al. [48] indicated negative impacts of biochar on aggregate formation and stability, likely influenced by the type, rate, and soil texture involved [49]. The CK MWD decreased by 11.80–20.29% compared to FK, mainly due to the frequent wet–dry cycles experienced with CI, which impact the pore structure within and between aggregates. These cycles cause aggregates to expand and contract, altering particle size distribution [50]. Overall, our results suggest that biochar application can help mitigate the adverse effects of CI on soil structure stability.

The soil pore structure shapes its biophysical and biogeochemical environment, with connected pores enhancing the exchange of air, water, and nutrients [51,52]. This study found that VF and CP were significantly positively correlated with MWD, Eh, CEC, SOC, and TN, while showing a negative correlation with density. This highlights the role of pore structure in influencing soil’s physical and chemical properties, including nutrient and organic carbon distribution, adsorption, retention, and release within soil aggregates [53]. Soil pore throat structure is a crucial component of soil porosity, significantly affecting water retention, nutrient cycling, and gas exchange [54]. Correlation analysis indicated strong associations between pore throat microstructure (TTN, SAR, EqR) and both CP and VF, with correlation coefficients of 0.86, 0.53, and 0.51 for CP, and 0.83, 0.66, and 0.60 for VF, respectively (Figure 7A). Under CA treatment, soil microstructure (VF, CP, TTN, SAR, and EqR) significantly increased (Table 1), consistent with findings by Feng et al. [51], which reported that biochar from pyrolyzed corn residue improved micropore systems, enhancing porosity, connectivity, and pore throats. This effect likely arises from the biochar’s porous structure (0.43 m^3^/g) and large surface area (682 m^2^/g), which improve the pore structure of soil [55,56]. Additionally, biochar acts as a long-term stable cementing agent that promotes the formation of macroaggregates from soil micro-particles, reducing bulk density by 0.97–1.37% [57,58]. Conversely, soil microstructure decreased significantly under FK compared to CK, potentially due to prolonged flooding that saturates soil voids. In contrast, CI’s wetting and drying cycles modify pore throat structures, enhancing porosity and complexity [59].

### 3.2. Impact of Soil Microstructure on Soil Microbial Composition and Function with Biochar and Water-Saving Irrigation

Previous research has focused on the relationships between microbial diversity, greenhouse gas emissions, and soil chemical properties [5]. However, this is the first study of the impact of soil microstructure on microbial communities in paddy soil. Spearman correlation analysis was conducted to explore the relationships among dominant bacterial and fungal families, soil physicochemical properties (MWD, density, pH, Eh, CEC, SOC, TN, C/N LMA, C/N MIA, C/N CLA), and soil microstructure (VF, CP, TTN, SAR, EqR), as shown in Figure 7. *Mycobacteriaceae* and *Streptomycetaceae* were positively correlated with SAR (0.62 and 0.51) and EqR (0.64 and 0.51), with relative abundances 4.39–5.10% and 1.71–16.38% higher in CA than in CK (Figure 5). This demonstrates that biochar addition increases SAR and EqR (Table 2), leading to a more complex microstructure that influences gas exchange and water distribution, thereby impacting microbial abundance [60,61]. Such findings highlight the importance of soil microstructure in providing habitats for microorganisms, supporting their abundance, diversity, and activity [1]. *Nocardioidaceae*, which enhances soil aggregation, aeration, and water retention, showed significant positive correlations with Eh, VF, CP, TTN, SAR, and EqR (correlation coefficients of 0.69, 0.50, 0.58, 0.69, 0.73, and 0.77) (Figure 7A). During the JS, *Nocardioidaceae* abundance in CA was 11.61% higher than in CK, while FK was 19.60% lower, which explains the increase in soil microstructure under biochar application and CI (Table 1 and Table 2). *Anaeromyxobacteraceae,* as anaerobic bacteria in paddy soil, were more abundant under FK than CK and showed significant negative correlations with Eh, TTN, SAR, and EqR (0.79, 0.68, 0.69, 0.81), reflecting higher Eh, TTN, SAR, and EqR values in CK compared to FK (Figure 1, Table 2). CI-induced increases in microstructure expose more surface area to air, raising Eh levels and suppressing anaerobic bacterial growth [62]. Additionally, C/N MIA was positively correlated with *Rhizobiaceae*, *Comamonadaceae*, and *Anaeromyxobacteraceae* (correlation coefficients of 0.49, 0.66, and 0.55), while C/N CLA was correlated with *Nitrobacteraceae* (0.47). These microbes, involved in nitrogen and carbon cycling and organic matter decomposition, predominantly form associations in aggregates > 53 µm. This underscores the essential role of soil aggregate structure and stability in supporting soil organism abundance, diversity, and function [23]. *Trichocomaceae* are essential to soil ecosystems, contributing to organic matter decomposition, nutrient cycling, and soil aggregation [63]. Our study revealed a significant negative correlation between *Trichocomaceae* abundance and soil properties such as MWD, pH, CEC, SOC, and TN (Figure 7B), with *Trichocomaceae* levels notably lower in CA than in CK (Figure 5B). This is likely due to biochar’s alkaline properties (pH 9–11) and its abundance of alkaline functional groups (e.g., carboxyl and phenolic hydroxyl), which raise soil pH [64]; but *Trichocomaceae* thrive in acidic environments, and the elevated pH in CA treatments may inhibit their growth.

To further explore the relationships between dominant bacterial and fungal families, metabolite levels, and environmental factors, RDA was conducted based on soil physicochemical properties and microstructure characteristics (Figure 7). The RDA results indicated that these factors could explain 70.30% of the soil bacterial community composition variation, with density, Eh, C/N LMA, C/N MIA, and soil microstructure being the dominant environmental factors (Figure 7C). The FK samples did not show significant correlations with these indicators, whereas the CK samples strongly correlated with soil microstructure, suggesting that CI influences soil microstructure. Additionally, *Sphingomonadaceae*, *Streptomycetaceae*, and *Micrococcaceae* exhibited significant positive correlations with SAR and EqR, showing higher abundance in the TS-CK samples, indicating that CI impacts soil microstructure and microbial composition, although this effect decreases over time. Compared to CK, CA significantly increased the abundance of *Streptomycetaceae* (Figure 7A) due to two main factors: (1) Biochar addition significantly enhanced the soil microstructure. Compared to CK, CA increased EqR, SAR, TTN, VF, and CP by 1.47–9.61%, 7.50–25.21%, 41.55–45.99%, 61.12–73.04%, and 46.36–93.75%, respectively (Table 1 and Table 2), thereby expanding microbial habitats and providing refuges for microorganisms [65]. (2) Biochar can improve the soil’s physical and chemical environment. Compared to CK, CA increased CEC, MWD, SOC, and TN by 22.14–25.06%, 42.24–56.61%, 22.98–56.5%, and 9.41–87.83%, respectively (Figure 1 and Figure 2), enhancing soil structure stability and carbon storage, which in turn promoted microbial community diversity [66,67]. The abundance of *Anaeromyxobacteraceae* was significantly positively correlated with C/N LMA under TS-FK, indicating its prevalence in FI environments with high C/N ratios within larger soil aggregates (>2000 μm), as FI provides hypoxic conditions favorable for its growth. *Comamonadaceae* demonstrated significant positive correlations with C/N MIA, reflecting its distribution in soil aggregates (53–250 μm) with high C/N ratios. In CA treatment, *Comamonadaceae* abundance increased by 32.96–68.06% compared to CK, likely due to biochar’s enhancement of C/N in MIA (Figure 2D), enriching the organic environment and supporting bacterial growth. RDA also showed that environmental factors explained 52.55% of fungal community composition variation, indicating a relatively weaker effect of these factors on fungi (Figure 7D). Future studies should investigate additional, unmeasured influences such as climatic variability.

Soil metabolites play a crucial role in biogeochemical cycles and nutrient cycling, fundamental to soil health and aggregate formation [68]. C/N LMA, C/N MIA, along with EqR, showed significant correlations with Level 2 and Level 3 metabolites (Figure S4), highlighting the importance of soil aggregate C/N ratios and throat equivalent radius in soil microstructure for understanding microbial functions and gene activity. RDA results indicated that environmental factors could explain 94.46% and 83.62% of the variance in Level 2 and Level 3 metabolites, respectively (Figure S4C,D). The results indicate that soil metabolites play an essential role in C/N MIA (53–250 μm), aligning with Totsche et al., [69]. Their study demonstrated that soil microaggregates (<250 μm) are core structures supporting vital soil functions, such as carbon storage and stabilization, microbial habitat diversity, nutrient and trace element cycling, and water retention [70]. In TS, carbohydrate metabolism pathways were significantly elevated in CA compared to CK (Figure 6), whereas this difference was less pronounced in JS, likely due to CI exposing soil to varied moisture and oxygen conditions, leading to biochar aging and degradation over time [71,72]. Biochar contributes to improved soil porosity, aeration, and stability [34], enhancing organic carbon and nutrient levels [73], which ultimately supports microbial activity and promotes carbon metabolism. Soil metabolites at KEGG Levels 2 and 3 were highly correlated with FK, while CA significantly enhanced amino acid metabolism, especially in ketone body synthesis and degradation. This suggests that biochar enhances these metabolic pathways by improving the soil microstructure, increasing the MWD and CEC, and fostering soil resilience under CI. From a metabolomics perspective, this demonstrates biochar’s ability to counteract the negative impacts of CI by promoting beneficial metabolic processes in the soil. In conclusion, soil microbiome function largely depends on the influence of environmental factors on soil aggregates and their microstructure, which biochar application can beneficially modulate for CI.

## 4. Materials and Methods

### 4.1. Experimental Site and Design

**Figure 8:**
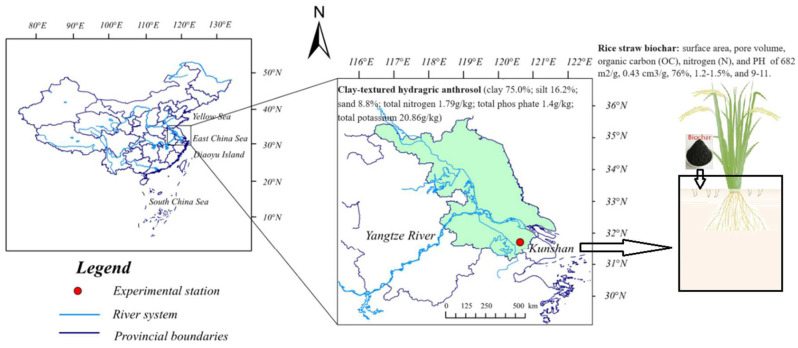
Basic information about the study area and biochar.

The experiment was conducted at the Kunshan Experimental Station, part of the State Key Laboratory of Hydrology-Water Resources and Hydraulic Engineering of Hohai University, located at 34°15′21″ N, 121°05′22″ E. The field site features a typical subtropical southern monsoon climate, with average annual temperatures of 18.40 °C, and average yearly precipitation and evaporation measuring 3.57 mm and 2.69 mm, respectively [74]. The biochar was purchased from Zhengzhou Luhang Purification Materials Company Limited (Zhengzhou, China), where it was produced via slow pyrolysis of rice straw at 600 °C in a low-oxygen environment. The detailed basic information on soil and biochar is shown in Figure 8.

Topsoil (0–20 cm) from rice fields cultivated for over 40 years was collected in 2022, air-dried, sieved (<5 mm), and refilled into cylindrical pots (35 cm in diameter, 42 cm in height) with 35 kg of soil per pot, maintaining the local bulk density of 1.30 g/cm. A mobile greenhouse was employed to mitigate the impact of rainfall. The experimental setup involved planting three rice seedlings per hole of the Nanjing 46 variety, with the cultivation period covering the entire growth cycle of the crop (from 7 May to 26 October). The study included three treatments: CI without biochar (CK), CI with 40 t/hm^2^ biochar [75,76] (CA), and FI without biochar (FK). Before planting, biochar was evenly mixed into the top 30 cm of soil to ensure proper integration. Each treatment was repeated 6 times, resulting in a total of 18 pots. In the CI treatment, water depth was regulated to 5–25 mm during the re-greening stage (RS), with no standing water allowed during other stages. In contrast, the FI treatment water depths are 30–50 mm after transplanting, except during the late-tillering stage (TS) and milky ripening stages (MR) [5]. The fertilization schedule for the experiment was as follows: The fertilization schedule for the experiment involved applying compound fertilizer (N:P:K = 19%:7%:12%) and urea (N ≥ 46.2%) separately on 4 July, with nitrogen application rates of 84.00 kg/ha and 103.95 kg/ha, respectively. Subsequently, on 25 July, the tillering fertilizer contributed 69.30 kg/hm^2^ of nitrogen; on 20 August, the panicle fertilizer provided an additional 55.44 kg/hm^2^ of nitrogen.

### 4.2. Soil Sampling and Properties

Soil samples were collected at two stages in 2022, including the TS and the jointing and booting stages (JS). To minimize disturbance to the soil structure, we collected samples from the 0–20 cm layer using PVC soil columns (50 mm in diameter and height). These columns were wrapped in plastic film and placed in shockproof bags to maintain their integrity during transport. CT scanning was performed to capture images of the soil microstructure. At each pot, some undisturbed soil samples were used to assess soil aggregate stability and density. The remaining samples were sieved (<2 mm), transported to the lab, and divided into two portions. One portion was stored at −80 °C for DNA extraction, while the other was kept at 4 °C for chemical analysis of the SOC and TN.

### 4.3. Determination of Physical and Chemical Properties of Soil

Using a soil aggregate analyzer, 50 g of fresh soil sample was divided into four size fractions: large macroaggregates (LMA, >2000 μm), small macroaggregates (SMA, 250–2000 μm), microaggregates (MIA, 53–250 μm), and a silt and clay fraction (CLA, <53 μm). Aggregates from each sieve were sequentially collected, rinsed into aluminum containers, dried at 60 °C, weighed, and measured for mean weight diameter (MWD). Soil density was measured using a stainless-steel cylinder with a cutting edge pressed into the topsoil. Soil pH was determined using a 1:2.5 soil-to-water ratio. Cation exchange capacity (CEC) was measured using the Hexamminecobalt trichloride solution-spectrophotometric method. The oxidation-reduction potential (Eh) of soil samples was determined in situ before each sampling using an ORP30p soil redox potential meter (Shanghai, China). SOC was determined via the potassium dichromate oxidation spectrophotometry method. TN was determined by the modified K9840 Kjeldahl method.

### 4.4. CT Scanning and Image Processing

Soil columns of PVC were X-ray scanned using SR-u CT (beamline BL13W1, operating at 23 KeV) at the Shanghai Synchrotron Radiation Facility (Shanghai, China). A PCO2000 CCD camera captured 1765 X-ray projections, reconstructed into tomograms with a 25 μm pixel resolution. A 512 × 512 × 512-pixel region of interest (ROI) was meticulously chosen at the image center to minimize edge effects. Image processing, reconstruction, visualization, and quantification were conducted using ImageJ version 1.52 (National Institutes of Health) and Avizo 2022 software (Massachusetts, USA) [77]. The ROI was normalized using “Interactive Thresholding”, followed by noise reduction with a median filter to enhance image clarity. The connectivity porosity of macroaggregates was analyzed using the “Axis Connectivity” module, and pore throat determination employed the “Generating Pore Network Model” tool. The fractal dimension was calculated using the “Fractal Dimension” tool. Pore data were exported to Excel for further analysis. Pore properties (volume fraction, connected porosity, area, and pore equivalent diameter) and throat properties (number, surface area, channel length, and equivalent radius of throats) were all obtained using the “Label Analysis” module of Avizo software [51].

### 4.5. Determination of Soil Microbial Community Diversity, Composition, and Metabolite Analysis

Total DNA from soil samples was extracted using the Mag-Bind Soil DNA Kit (Georgia, USA). The purity, concentration, and integrity of DNA extracted from soil samples were determined using a Qubit^®^ 2.0 fluorometer (Massachusetts, USA), followed by assessment through 1% agarose gel electrophoresis. Approximately 1 μg of DNA per sample was used to construct 350 bp libraries with the DNA Library Prep Kit (Massachusetts, USA), incorporating barcodes. DNA fragments underwent end-polishing, A-tailing, and adapter ligation, followed by PCR amplification. Libraries were purified with the AMPure XP system and evaluated using an Agilent 2100 Bioanalyzer (California, USA). Clustering was performed with the cBot Cluster Generation System, and paired-end sequencing (150 + 150 bp) was conducted on an Illumina NovaSeq 6000 platform (Illumina Inc., San Diego, CA, USA) at Wekemo Tech Co., Ltd. (Shenzhen, China). Raw sequencing reads were thoroughly processed, including filtering, de-duplication, denoising, and host sequence removal [78]. The KneadData pipeline (v0.7.4) was used for quality control and host sequence removal, processing raw sequencing data to generate clean sequences suitable for downstream analyses. This process used Trimmomatic (v0.39) for quality control and Bowtie2 (v2.3.5.1) for host sequence removal, with quality assessment using FastQC (v0.11.9) both before and after processing to verify the effectiveness of our quality control measures [79]. For specific procedures, please refer to the DNA extraction methods in Zhang et al. [71]. The metabolic pathways and enrichment analysis of these pathways were conducted using the Kyoto Encyclopedia of Genes and Genomes (KEGG) database. Microbial diversity was assessed using Chao1, Simpson, and Shannon indices based on OTUs, with the respective formulas provided by Cui et al. [80].

$$\begin{array}{l} Chao1=S_{obs}+\frac{n_{1}(n_{1}-1)}{2(n_{2}+1)}\\ Shannon=-\sum_{i=1}^{S_{obs}}\frac{n_{i}}{N}\ln\frac{n_{i}}{N}\\ Simpson=\frac{\sum_{i=1}^{S_{obs}}n_{i}(n_{i}-1)}{N(N-1)} \end{array}$$

*S_obs_* denotes the count of observed OTUs; *n*_1_, *n*_2_, and *n_i_* represent the number of OTUs consisting of one sequence, two sequences, and the *i*th number of sequences, respectively; *N* represents the total number of sequences [81,82,83].

### 4.6. Statistical Analysis

Data were collated using Microsoft Excel 2021 (Washington, USA). Treatment group comparisons were performed using one-way analysis of variance (ANOVA) in SPSS 23.0 (New York, USA) followed by post-hoc least significant difference (LSD) tests at a significance level of *p* < 0.05. Bonferroni-adjusted significance thresholds were used where appropriate, particularly for analyses involving multiple dependent variables, to minimize type I error rates. Bar and line charts were created using Origin 9.1 (OriginLab Corporation, Northampton, MA, USA), and clustering heatmaps were generated using the R Project for Statistical Computing (http://www.R-project.org). Redundancy analysis (RDA) was conducted using Canoco 5.0 for Windows (Microcomputer Power, Ithaca, NY, USA).

## 5. Conclusions

The connection between soil structure and microbiome function is crucial for agricultural ecosystems. However, to our knowledge, this is the first study to explore the interactions between soil microstructure, soil physicochemical properties, and microbial composition and function under different irrigation regimes and biochar applications in paddy fields. CA and FK significantly raised the proportion of aggregates (>250 μm), leading to a 42.24–56.61% and 11.80–20.29% increase in MWD compared to CK, indicating that biochar application can help mitigate the adverse effects of CI on soil structure stability. Soil microstructure (VF, CP, TTN, SAR, EqR) increased significantly under CA but decreased under FK compared to CK. Notably, VF and CP showed a positive correlation with MWD, Eh, CEC, SOC, TN, and pore throat microstructure indices while exhibiting a negative correlation with soil density, highlighting the significant role of microstructure in influencing the physical and chemical properties of soil. *Nocardioidaceae* abundance showed a strong positive correlation with Eh and soil microstructure metrics. Conversely, *Anaeromyxobacteraceae*, more prevalent under FK than CK, correlated negatively with Eh, TTN, SAR, and EqR (0.79, 0.68, 0.69, 0.81), suggesting that the increased air exposure from CI reduced conditions favoring anaerobic bacteria. RDA indicated that the soil physicochemical properties and microstructure explained 70.30%, 52.55%, 94.46%, and 83.62% of the variability in soil bacterial and fungal communities, as well as metabolite profiles at Levels 2 and 3, respectively. CK was strongly associated with the soil microstructure, whereas FK showed no significant correlation with these indicators. CA substantially improved the soil microstructure and the physical-chemical environment, supporting higher abundances of *Mycobacteriaceae* (5.10%), *Streptomycetaceae* (1.71%), and *Nocardioidaceae* (11.61%), and enhanced the functional metabolic pathways, particularly amino acid metabolism and ketone body synthesis and degradation. This suggests that soil microbiome composition and function are largely shaped by environmental impacts on soil microstructure, which biochar application can beneficially modulate for CI. Future studies should investigate the relationships between soil microporosity structure and soil microorganisms under different types of biochar or analyze the long-term effects.

## Figures and Tables

**Table 1:** Microporosity of soil samples under different water-carbon management strategies by SR-μCT analysis.

	Volume Fraction	Equivalent Diameter (μm)	Area (µm^2^)	Connected Porosity	Fractal Dimension
TS-CK	6.37% ± 0.12% d	103.79 ± 3.42 a	137,114.59 ± 16,469.29 a	4.17% ± 0.92% cd	2.47 ± 0.01 a
TS-CA	11.02% ± 1.03% b	114.67 ± 18.4 a	326,084.22 ± 227,949.58 a	8.08% ± 1.33% ab	2.5 ± 0.02 a
TS-FK	5.83% ± 0.16% d	84.74 ± 17.28 a	98,976.97 ± 25,775.96 a	3.7% ± 1.73% d	2.5 ± 0.09 a
JS-CK	7.43% ± 0.31% c	83.61 ± 26.95 a	161,231.53 ± 113,774.71 a	6.04% ± 0.19% bc	2.49 ± 0.06 a
JS-CA	11.97% ± 0.58% a	125.17 ± 70.94 a	401,916.56 ± 304,676.99 a	8.84% ± 1.6% a	2.5 ± 0.1 a
JS-FK	6.02% ± 0.22% d	99.82 ± 13.53 a	148,756.48 ± 8843.36 a	2.15% ± 1.06% d	2.42 ± 0.04 a

Note: Different lowercase letters indicate significant differences at the 0.05 level in the same column.

**Table 2:** Pore throats parameters of interconnected pores in the pore system.

	Total Throat Number	Surface Area (µm^2^) × 100	Equivalent Radius (µm)	Channel Length (µm)
TS-CK	5219 ± 766 c	46,824.49 ± 5888.94 b	97.68 ± 4.87 b	450.68 ± 80.72 a
TS-CA	7619 ± 781 ab	50,335.27 ± 6330.48 ab	99.13 ± 4.94 b	478.92 ± 85.78 a
TS-FK	1476 ± 257 d	32,764.93 ± 4120.72 c	80.39 ± 4.01 c	445.98 ± 79.88 a
JS-CK	6260 ± 1452 bc	47,890 ± 6022.95 b	100.15 ± 4.99 b	447.26 ± 80.11 a
JS-CA	8836 ± 1123 a	59,962.5 ± 7541.26 a	109.78 ± 5.47 a	516.79 ± 92.56 a
JS-FK	1937 ± 517 d	35,344.49 ± 4809.52 c	82.87 ± 4.76 c	404.33 ± 74.72 a

Note: Different letters following values indicate a significant difference between treatments (*p* < 0.05).

## Data Availability

Data will be made available on request.

## Supplementary Materials

The following supporting information can be downloaded at https://www.mdpi.com/article/10.3390/plants14101498/s1: Figure S1. ANOVA of Shannon’s index, Chao1, and Simpson’s index of soil microbial diversity in each treatment. (A,C,E) represents bacteria, (B,D,F) represents fungi. Different lowercase letters indicate significant differences at 0.05 level between different treatments; Figure S2. Relative abundance of the phyla level bacteria (A) and fungi (B) with the highest abundance; Figure S3. Gene abundance of KEGG metabolic pathways, cluster heatmap of level 1 and level 2; Figure S4. Pearson correlation coefficients of the relationships between dominant level 2 pathway genes (A) and level 3 pathway genes (B), and soil physicochemical (MWD, density, pH, Eh, CEC, SOC, TN, C/N LMA, C/N MIA, C/N CLA) and soil microstructure (VF, CP, TTN, SAR, EqR), respectively (* *p* < 0.05). Redundancy analysis (RDA) curves of the dominant level 2 metabolites information (C), and level 3 metabolites information (D). Red arrows indicate environmental variables; Blue arrows indicate species; Symbols represent the soil samples. * *p* < 0.05; Table S1. Multivariate ANOVA for the effects of biochar (B), Irrigation (I), and Stage (S) on Soil physicochemical properties, micro-pore structure, microbial diversity, and abundance; Table S2. Gene abundance of KEGG level 1 pathway Unit(%).
