# Supplementary material for: Microstructure and Microorganisms Alternation of Paddy Soil: Interplay of Biochar and Water-Saving Irrigation
Source: Plants (Basel). 2025 May 16;14(10):1498. doi: 10.3390/plants14101498 (PMC12114665; doi:10.3390/plants14101498)
Supplement: Supplementary file 1 [file plants-14-01498-s001.zip › plants-3550735-supplementary.pdf]

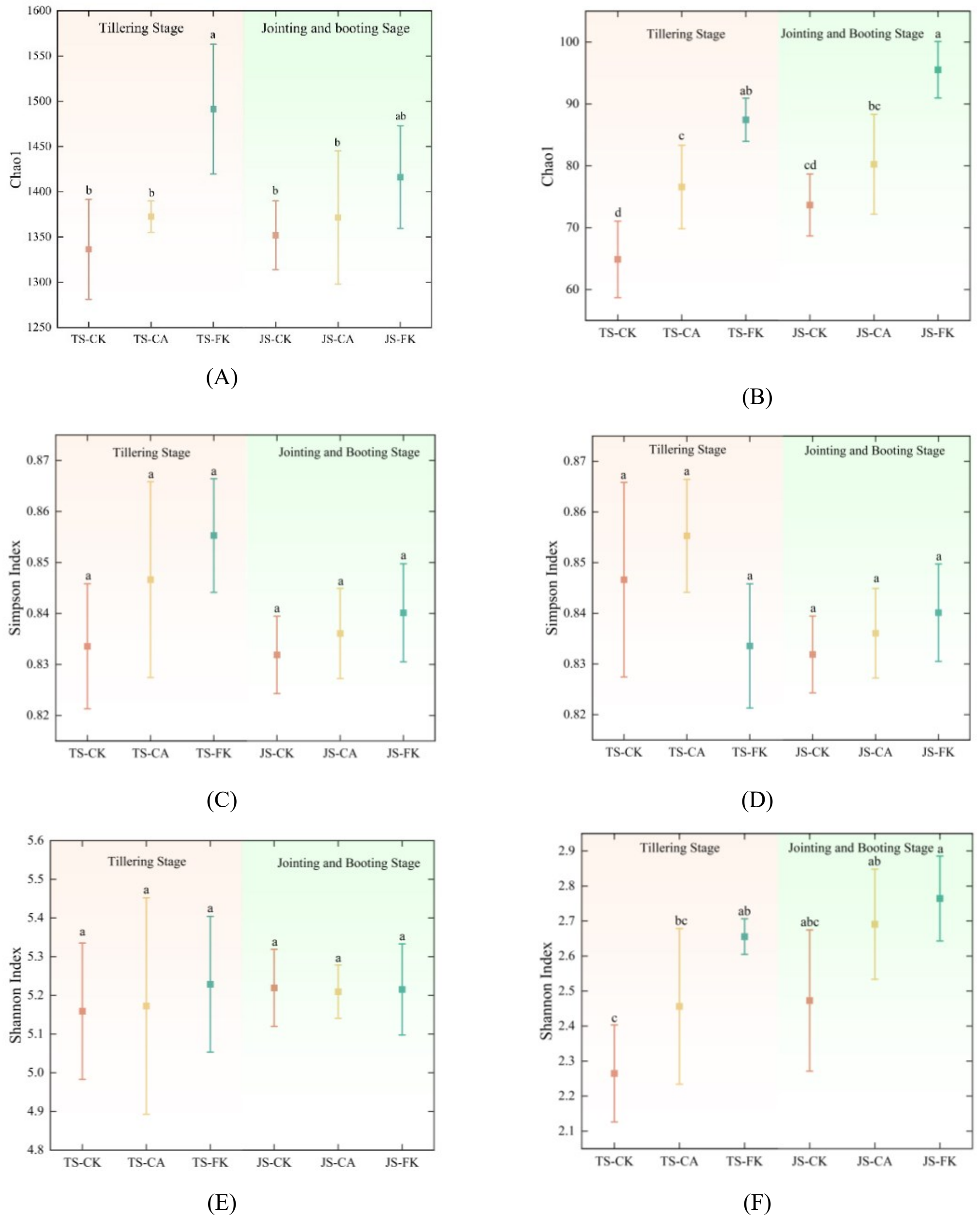

Figure S1. ANOVA of Shannon's index, Chao1, and Simpson's index of soil microbial diversity in each treatment. (A), (C), (E) represents bacteria, (B), (D), (F) represents fungi Different lowercase letters indicate significant differences at 0.05 level between different treatments.

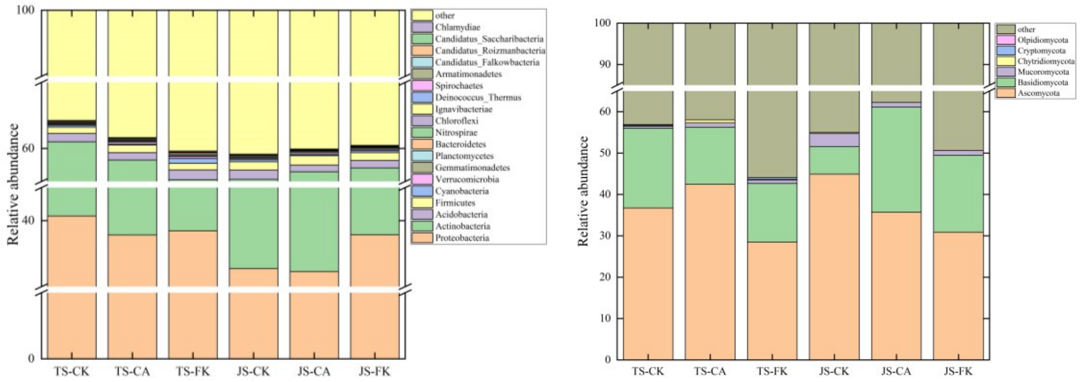

(A) (B)

Figure S2. Relative abundance of the phyla level bacteria (A) and fungi (B) with the highest abundance.

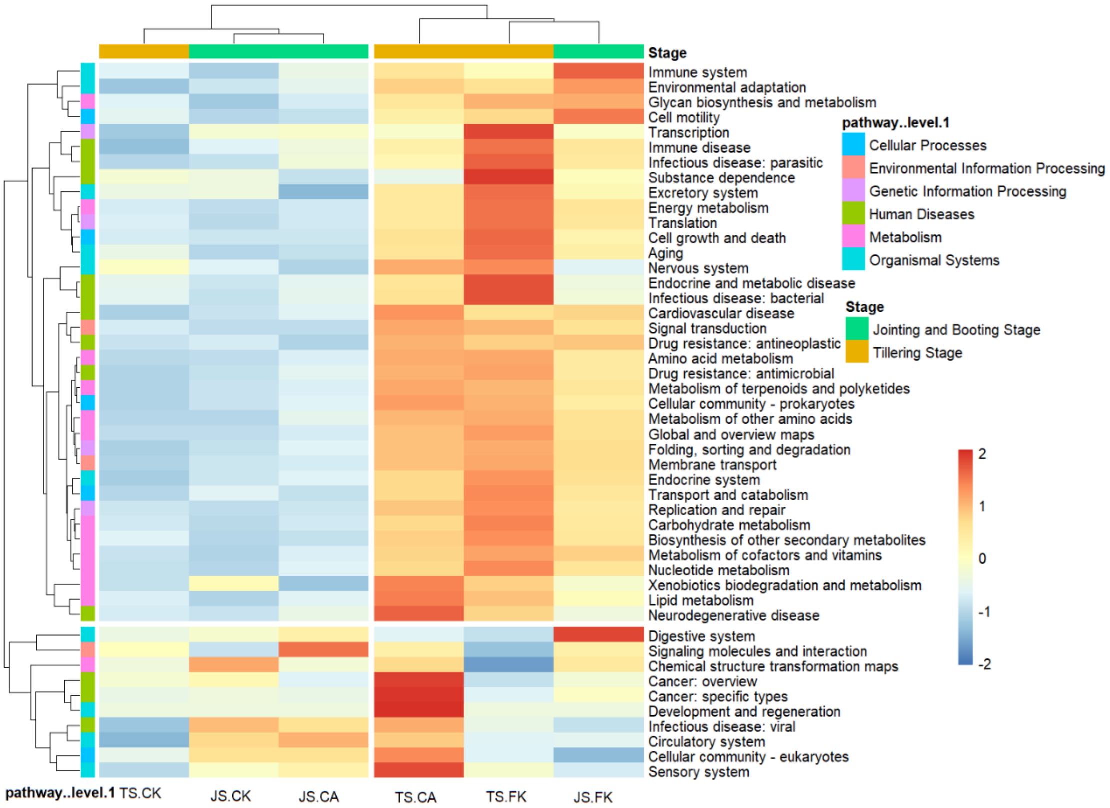

Figure S3. Gene abundance of KEGG metabolic pathways, cluster heatmap of level 1 and level 2.

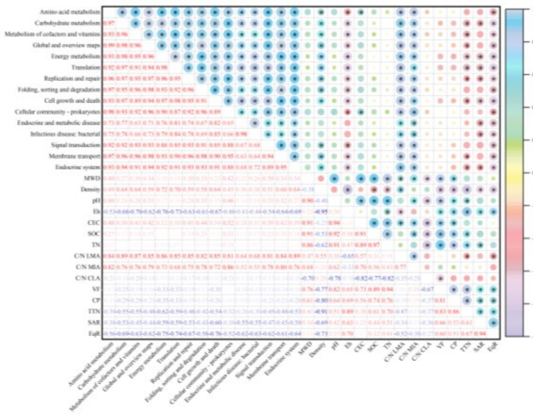

(A)

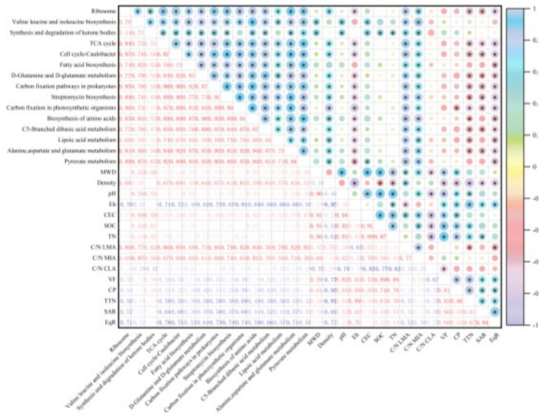

(B)

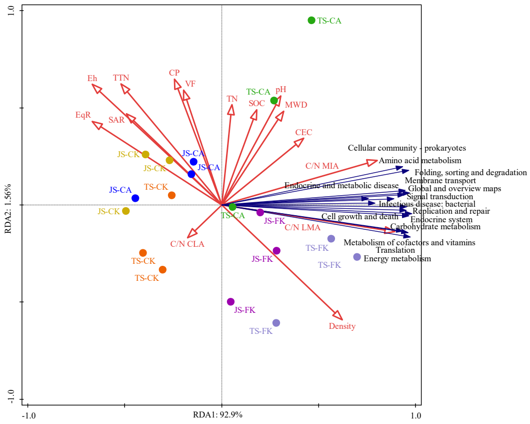

(C)

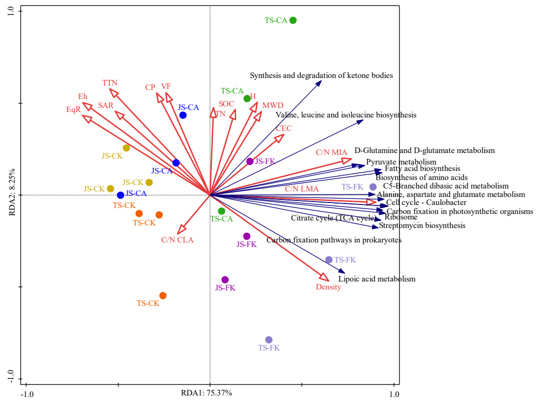

(D)

Figure S4. Pearson correlation coefficients of the relationships between dominant level 2 pathway genes (A) and level 3 pathway genes (B), and soil physicochemical (MWD, density, pH, Eh, CEC, SOC, TN, C/N LMA, C/N MIA, C/N CLA) and soil microstructure (VF, CP, TTN, SAR, EqR), respectively (*P < 0.05). Redundancy analysis (RDA) curves of the dominant level 2 metabolites information (C), and level 3 metabolites information (D). Red arrows indicate environmental variables; Blue arrows indicate species; Symbols represent the soil samples. *p < 0.05.

Table S1. Multivariate ANOVA for the effects of biochar (B), Irrigation (I), and Stage (S) on Soil physicochemical properties, micro-pore structure, microbial diversity, and abundance

Properties		P (F) value						
		Biochar (B)	Irrigation (I)	Stage (S)	B×I	B×S	I×S	B×I×S
Soil physicochemical properties	MWD	56.57**	17.71**	0.53	38.32**	18.69**	10.12*	17.18**
	Density	9*	160**	0.14	131.42**	4.46*	49.14**	56.5**
	pH	110.17**	18.34*	0.35	84.27**	49.35**	5.91*	45.74**
	Eh	7.93*	787.87**	0.012	693.59**	10.82*	1786.05**	1856.08**
	CEC	255.43**	18.06*	1.29	65.36**	273.61**	84.60**	164.59**
	SOC	245.13**	6.43*	0.16	125.53**	74.76**	3.08	50.74**
	TN	269.26**	0.77	0.11	161.15**	114.18**	9.05*	120.99**
	C/N LMA	18.67**	34.96**	3.08	19.92**	13.715**	24.48**	23.18**
	C/N SMA	0.34	0.52	0.30	0.85	0.14	0.27	0.34
	C/N MIA	20.70**	14.40**	1.59	9.91*	16.41**	7.91**	8.87*
	C/N CLA	24.99**	2.71	0.65	14.85**	42.48**	7.50**	15.95**
Micro-pore structure	Volume Fraction	104.62**	13.57*	0.35	127.37**	58.52**	33.04**	82.74**
	Equivalent Diameter (um)	1.59	0.02	0.01	1.53	0.62	1.05	0.73
	Area (μm^2)	4.25	0.59	0.34	4.74*	1.25	0.62	1.68
	Connection porosity	20.46**	7.56*	0.08	24.67**	10.25*	6.14*	13.27**
	Fractal dimension	0.43	0.43	0.46	0.72	0.17	1.21	0.80
Throats structure	Total Throats Number	14.31*	70.74**	0.27	76.71**	6.55*	24.64**	35.13**
	Surface Area (μm^2)	3.82	3.61	3.60	6.51*	2.56	5.02*	6.3*
	Equivalent radius (μm)	1.17	7.72*	5.15	7.02*	3.52	11.44*	11.58**
	Channel Length (μm)	1.20	0.06	0.32	0.68	0.43	0.07	0.32
Bacteria	Chao1	1.12	10.48*	0.35	6.38*	0.35	4.67*	3.10
	Shannon	0.02	0.08	0.16	0.10	0.08	0.06	0.08
	Simpson	0.58	0.10	2.34	0.70	2.13	0.82	1.65
Fungi	Chao1	5.19*	36.41**	1.75	17.33**	2.97	23.46**	10.04**
	Shannon	3.03	14.73*	3.64	5.67	2.72	7.47*	4.12*
	Simpson	0.39	1.11	3.34	0.71	1.10	1.43	1.11

Note: *p < 0.05, **p < 0.01.

Table S2. Gene abundance of KEGG level 1 pathway Unit(%)

	Cellular Processes	Environmental Information Processing	Genetic Information Processing	Human Diseases	Metabolism	Organismal Systems
TS-CK	5.21±0.01bc	1.97±0.05a	11.17±0.14a	4.76±0.06ab	73.67±0.11ab	3.21±0.1a
TS-CA	5.29±0.08b	2.07±0.06a	11.11±0.03a	4.82±0.04ab	73.54±0.18ab	3.17±0.12a
TS-FK	5.46±0.05a	2.02±0.07a	11.45±0.18a	4.73±0.08ab	73.03±0.04b	3.31±0.14a
JS-CK	5.13±0.07bc	2±0.03a	11.2±0.28a	4.88±0.12a	73.58±0.41ab	3.21±0.1a
JS-CA	5.09±0.06c	1.98±0.04a	11.22±0.16a	4.85±0.19ab	73.76±0.31a	3.1±0.05a
JS-FK	5.53±0.2a	2.06±0.04a	11.24±0.17a	4.6±0.15b	73.37±0.52ab	3.2±0.11a

Note: Different lowercase letters indicate significant differences at 0.05 level in the same column.
